# High-Frequency and High-Current Transmission Techniques for Multiple Earth Electrical Characteristic Measurement Systems Based on Adaptive Impedance Matching through Phase Comparison

**DOI:** 10.3390/s24103110

**Published:** 2024-05-14

**Authors:** Kuiyuan Zhang, Shulin Yang, Meng Wang, Rongbo Zhang

**Affiliations:** 1 School of Geophysics and Information Technology, China University of Geosciences Beijing, Beijing 100083, China; zhangkuiyuan@cugb.edu.cn (K.Z.); 2110220064@email.cugb.edu.cn (S.Y.); 2110220024@email.cugb.edu.cn (R.Z.); 2 Key Laboratory of Intraplate Volcanoes and Earthquakes, China University of Geosciences, Beijing 100083, China; 3 State Key Laboratory of Geological Processes and Mineral Resources, China University of Geosciences, Beijing 100083, China

**Keywords:** MECS, near-surface geophysics, high frequency and high current transmission, adaptive impedance matching, high-current precisely controllable reactor

## Abstract

With the increase in groundwater exploration, underground mineral resource exploration, and non-destructive investigation of cultural relics, high-resolution earth electrical characteristic measurement has emerged as a mainstream technique owing to its advantageous non-destructive detection capability. To enhance the transmission power of the high-frequency transmitter in high-resolution multiple earth electrical characteristic measurement systems (MECS), this study proposes a high-frequency, high-current transmission technique based on adaptive impedance matching and implemented through the integration of resonant capacitors, a controllable reactor, high-frequency transformers, and corresponding control circuits. A high-current precisely controllable reactor with a 94% inductance variation range was designed and combined with resonant capacitors to reduce circuit impedance. Additionally, high-frequency transformers were employed to further increase the transmission voltage. A prototype was developed and tested, demonstrating an increase in transmission current at frequencies between 10 and 120 kHz with a peak active power of 200 W. Under the same transmission voltage, compared to the transmission circuit without impedance matching, the transmission current increased to a maximum of 16.7 times (average of 10.8 times), whereas compared to the transmission circuit using only traditional impedance matching, the transmission current increased by a maximum of 10.0 times (average of 4.2 times), effectively improving the exploration resolution.

## 1. Introduction

The increase in groundwater exploration, underground mineral resource exploration, and non-destructive investigation of cultural relics has heightened the demand for efficient and high-resolution multiple earth electrical characteristic measurements of shallow subsurface targets [1,2,3,4]. Addressing the challenge of generating large currents under wide bandwidth conditions and ensuring that effective signals of various frequencies excite the secondary electric fields of subsurface electrical bodies at various depths and scales is essential to enhancing the resolution of this exploration method. It also offers a robust technical solution for investigating new artificial source electromagnetic exploration methods [4,5].

Under identical subsurface conditions, the higher the transmitting current from the electromagnetic source transmitter, the stronger the electromagnetic field [6]. Consequently, the signals reaching the receiver end are stronger, leading to more effective exploration [7]. Increasing transmission power is a common method for optimizing high-frequency artificial field source energy technologies. Reference [8] developed the EMT-48 KW transmitter, which features a transmission power of up to 48 kW and a maximum transmission voltage of 950 V, enabling it to effectively increase the transmission current. However, as power increases, the instrument becomes bulkier, more complex, and costlier. Reference [9] suggests that optimizing the wire structure can reduce the magnetic density of the wire current, and effectively reduce inductance, thereby increasing high-frequency transmission current. However, designing the wire structure is relatively complex and requires special customization. In [10], a solution was presented where multiple parallel-connected or thicker transmission cables were used to reduce resistance and inductance in the transmitting cable, thereby increasing high-frequency transmitting current. However, during field construction, thickening or parallel connection of transmission cables has increased equipment weight and construction difficulty, hindering effective progress. In [11], a multi-polarized field was generated by adjusting the distribution of current among three electrodes, resulting in multi-polarized data that provides comprehensive subsurface coverage and illumination. In [12], the electromagnetic field strength was enhanced by employing four sets of synchronized transmission systems that emitted artificial electromagnetic field sources to the east, south, west, and north to improve the SNR. Reference [13] proposed a dual-transmitter system that increases high-frequency transmission current based on the parasitic inductance effect. However, solely relying on the transmission method to improve exploration accuracy imposes higher demands on instrument precision, quantity, and control, which may hinder fieldwork realization. Furthermore, reference [14] employed an RLC series resonant full-bridge inverter high-power transmission circuit, effectively increasing the transmitted current by raising the transmission voltage based on the series resonance principle. Additionally, reference [15] employed the RLC-series resonance principle and multiple relays to control a network of seven resonant capacitors. By varying the connection of different capacitors to the circuit, the impedance of the transmission circuit was reduced, thereby increasing the transmission current of the transmitter. Compared to changing the transmission method, these two solutions require less instrument precision, quantity, and control, making them more suitable for fieldwork because they only utilize resonant capacitors to reduce the circuit’s reactance and increase the transmission current. However, this method fails to consistently increase transmission current across multiple frequencies and performs well only at specific frequencies.

Aligning with the current trend of developing lighter, more compact, highly integrated, and more stable transmitters, this study proposes a high-frequency, high-current transmission technique for electromagnetic transmitter systems based on adaptive impedance matching through phase comparison. Table 1 lists various techniques used for boosting high-frequency artificial field source intensity, including the one proposed in this study. Considering that the impedance of the transmission circuit rises rapidly as the transmission frequency increases, the technique proposed in this study focuses on lowering circuit impedance to effectively increase active power output rather than directly increasing the transmitter’s power output, thus making it more cost-effective. Unlike the methods proposed in references [9,10], the technique introduced in this study allows for the direct integration of the prototype into existing transmission circuits to enhance transmission current, making it better suited for field operations and leading to a more effective transmitted current. Moreover, the methods proposed in references [11,12,13] require higher instrument precision, quantity, and control as the transmission frequency increases; otherwise, the effectiveness improvement cannot be assured. However, the technique developed in this study, which involves impedance matching and increasing transmitted voltage, offers simpler control, thereby enabling enhanced exploration accuracy at transmission frequencies ranging from 10 kHz to 120 kHz. Furthermore, in contrast to [14,15], the technique proposed in this study integrates resonant capacitors, controllable reactors, high-frequency transformers, and corresponding control circuits to reduce transmission circuit impedance and significantly enhance fundamental frequency transmission signals across a broad frequency range with multiple transmission frequencies. Consequently, this technique provides a cost-effective and easily controllable solution to substantially increase transmission current at more frequency points and at higher and wider frequency bandwidths.

This study provides an assessment of the operational status of the serial resonant circuit in the transmission circuit and a detailed design and analysis of the resonant capacitors, high-current precisely controllable reactor, and circuit structure in a high-frequency, high-current transmission technique based on adaptive impedance matching. The focus is on the circuit structure of the phase-difference measurement and control modules, along with their respective control programs. Finally, a prototype is presented along with validations of its effectiveness and feasibility.

## 2. Transmission Circuit Analysis

Analysis of the transmission circuit composed of a long cable and a transmitter reveals that because the wavelength corresponding to the maximum frequency of 120 kHz is much larger than the length of the cable, lumped parameters can be used for circuit impedance analysis. When the transmitter excites high-frequency AC signals, the impedance of the transmission circuit is equivalent to a series connection between the resistance and inductance. The transmission circuit impedance primarily includes ground resistance, transmission cable resistance, and reactance. The ground resistance is only related to the soil resistivity and ground area; it changes with the working location but does not vary with frequency changes. Despite the skin effect causing an increase in resistance on the transmission cable, for a 100 m copper cable with a cross-sectional area of 10 mm^2^, the resistance on the cable is only approximately 0.85 Ω. The inductance of the cable can be calculated using Equation (1) [16].

(1) $$L=\frac{\mu_{0}l}{2\pi }\left(ln(\frac{2l}{r_{0}})-0.75\right)$$

**Figure 1:**
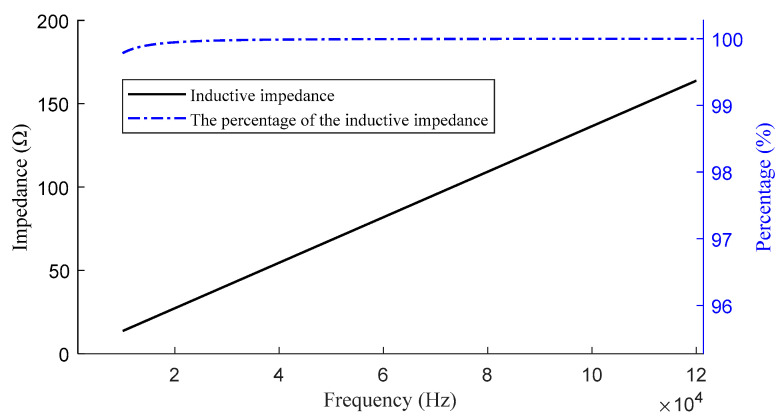
Impedance variation of the cable with frequency chart.

In Equation (1), *L* denotes the inductance, *l* denotes the length of the cable, *r*_0_ denotes the radius of the cable, and *μ*_0_ denotes the magnetic permeability of the cable, with its value being 4π × 10^−7^. Based on the above analysis, the magnitudes of the transmitter cable resistance and reactance on the cable, as well as the impedance distribution along the cable, can be determined, as shown in Figure 1. As depicted in Figure 1, the reactive impedance increases rapidly with frequency, resulting in a decrease in the magnitude of the emitted current.

To address these problems, impedance matching is required to convert the circuit into a series of resonant states. When the transmitter frequency is equal to the resonant frequency of the circuit, the circuit voltage is in phase with the circuit current, and the circuit impedance becomes resistive. At this point, the total impedance of the circuit is minimized, the transmitted current is maximized, and the transmitted active power is maximized.

**Figure 2:**
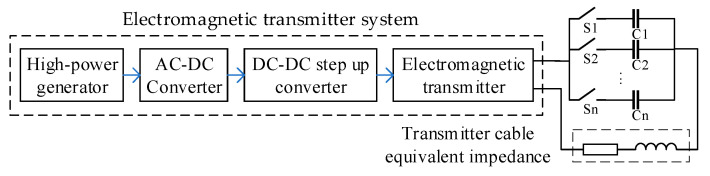
Transmission circuit with the traditional impedance matching technique.

An electromagnetic transmitter system involves rectifying the alternating current provided by a high-power generator, boosting it through DC-DC conversion, supplying it to the transmitter, and transmitting it using long power cables. The transmission circuit using the traditional impedance matching technique is illustrated in Figure 2. To enhance the transmitted current, the DC voltage supplied to the transmitter must be maximized, and impedance matching is achieved using resonant capacitors [14,15,17]. Compared to DC voltage boosting, the AC voltage can be boosted using a transformer, making the circuit and control simpler and more suitable for field operations. Relying solely on resonant capacitors for impedance matching fails to match multiple frequencies within the bandwidth and increases the device size as the bandwidth increases.

**Figure 3:**
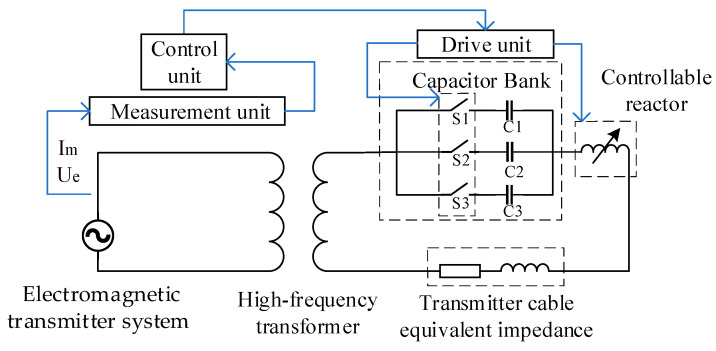
Transmission circuit with high-frequency, high-current transmission technique based on adaptive impedance matching.

Therefore, this article innovatively proposes a high-frequency, high-current transmission technique based on adaptive impedance matching, as shown in Figure 3. The system primarily comprises a measurement unit, a control unit, a drive unit, an impedance-matching unit, and a high-frequency transformer. The measurement unit measures the transmission frequency and phase difference between the output voltage *U*_e_ and current *I*_m_ at the transmitter end and transmits the measurement results to the control unit. Based on the measurement results, the control unit controls the drive unit to select the appropriate resonant capacitor in the impedance-matching unit and controls the high-current precisely controllable reactor to place the overall circuit in a resistive state. The high-frequency transformer amplifies the selected frequency, increases the transmission current at a specific frequency, and enhances the transmission power.

**Figure 4:**
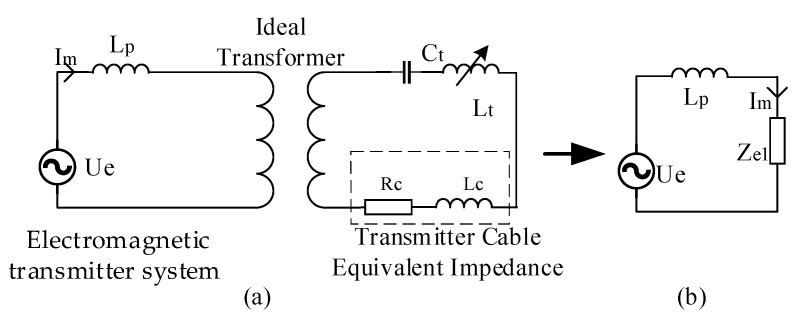
(**a**) Transmission circuit with high-frequency, high-current transmission technique based on adaptive impedance matching. (**b**) Equivalent circuit of the transmission circuit.

To design the high-current precisely controllable reactor and resonant capacitor groups used in impedance matching precisely, circuit equivalence must be achieved, as shown in Figure 4. Owing to the inherent structure of a high-frequency transformer, such as the arrangement of windings, insulation distance, and high operating frequency, an inevitable leakage inductance exists. With an increase in the operating frequency, the impact of leakage inductance on the efficiency of the high-frequency transformer gradually becomes more significant. For ease of calculation, the leakage inductance of the secondary winding of the transformer is equivalent to that of the primary side and is considered together with the leakage inductance of the primary winding, as shown in Figure 4a. Here, where *L*_p_ represents the sum of the leakage inductances of the primary and secondary windings of the high-frequency transformer, *C*_t_ and *L*_t_ denote, respectively, the resonant capacitor and the high-current-transmission controllable reactor selected at a specific frequency *f*_c_; *I*_m_ denotes the output current of the transmitter; and *R*_c_ and *L*_c_ denote the equivalent resistance and inductance of the transmission cable, respectively. Upon transforming the secondary circuit to the primary side, it can be equivalently represented by the equivalent circuit shown in Figure 4b, where *Z*_e1_ denotes the equivalent impedance on the primary side, and its magnitude is determined by Equation (2).

(2) $$Z_{e1}=\left(R_{c}+\left(\left(L_{t}+L_{c}\right)2\pi f_{c}-\frac{1}{2\pi f_{c}C_{t}}j\right)\right)n^{2}$$

Figure 4b demonstrates that when the reactive power in the circuit is zero, that is, the voltage phase of the circuit is synchronized with the current phase (*I_m_* is at its maximum), the total impedance of the transmission circuit is minimized, resulting in the maximum active power. According to the principle of conservation of active power, at this point, the resistive power consumption of the cable is maximized, leading to the maximum transmission current. Therefore, the measurement unit can detect the phase difference between *I_m_* and *U_e_*. When the phase difference is zero, the circuit is in resonance, achieving the maximum transmission current.

## 3. Technical Design Scheme for High-Frequency, High-Current Transmission Technique Based on Adaptive Impedance Matching

### 3.1. Analysis and Design of High-Current Precisely Controllable Reactor

**Figure 5:**
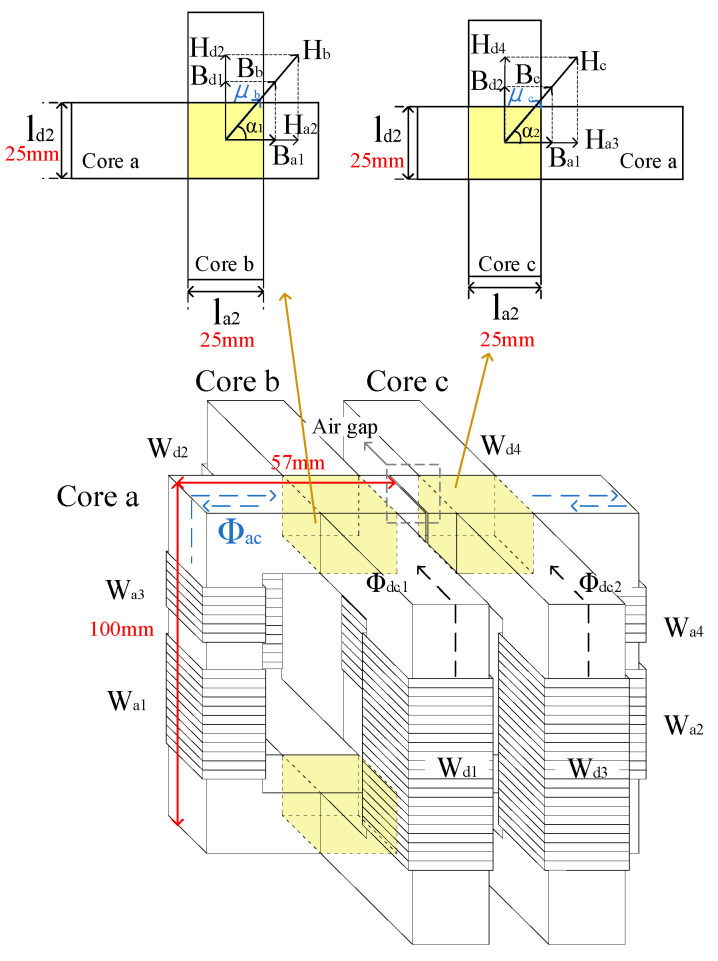
Structure of high-current precisely controllable reactor.

Traditional impedance-matching devices typically use only fixed resonant capacitors. With increases in the bandwidth and number of transmission frequencies, the number of resonant capacitors significantly increases. Therefore, a high-current precisely controllable reactor can effectively address the volume and cost issues associated with impedance-matching devices. The existing controllable reactors are primarily classified into five categories [18]: traditional, thyristor-controlled, magnetic-controlled, pulse-width modulation (PWM) controllable, and superconducting controllable reactors. Traditional controllable reactors exhibit discrete changes in the inductance values, and their mechanical structure, particularly the tap mechanism, is susceptible to damage during prolonged continuous adjustments. Thyristor-controlled reactors and PWM-controllable reactors incur higher costs under high-voltage conditions, and the introduction of power electronic devices may generate harmonics. The construction of cooling systems for superconducting controllable reactors is challenging [19,20,21]. Therefore, this study focuses on designing a magnetically controlled, high-current precisely controllable reactor, as depicted in Figure 5. The proposed device is a type of orthogonal DC magnetically controlled reactor that has evolved from parameter transformers [22]. It features multiple advantages, such as low harmonic content, continuous inductance magnitude adjustability, and a wide range of linear inductance variations [23,24]. The high-current precisely controllable reactor consists of six identical U-shaped magnetic cores made of manganese-zinc ferrite, as shown in Figure 5, with a saturation flux density (*B*_s_) of 510 mT at 25 °C. As illustrated in Figure 5, two of these cores form an AC core with four AC winding coils on their side pillars. The winding coils, W_a1_ and W_a2_, W_a3_ and W_a4_, are paired and connected in series, setting the number of turns for W_a1_ and W_a2_, and for W_a3_ and W_a4_, to *N*_a1_ = *N*_a2_ = 12 and *N*_a3_ = *N*_a4_ = 6, respectively. The remaining four cores are placed orthogonally to the AC magnetic cores, forming two DC magnetic cores, each with DC winding coils W_d1_, W_d2_, W_d3_, and W_d4_ wound on their side pillars. The DC winding coils are connected in series, and the paths of the AC and DC magnetic fluxes are shown as dashed lines in Figure 5. The DC winding coils are connected to the drive unit as control windings, whereas the AC winding coils are connected to the transmission circuit as operational windings. When excitation is separately applied to the AC and DC winding coils, the generated AC and DC magnetic fluxes form an orthogonal magnetic field at the intersection area of the cores. The magnetic field strength of the orthogonal part can be altered by controlling the magnitude of the DC, which effectively alters the magnetic permeability of the orthogonal part. This method results in a controllable inductance of the operational windings. Magnetically controlled, high-current precisely controllable reactors using orthogonal structures have the advantages of low harmonic content, predominantly linear inductance change, and small space occupation. Thus, the high-current precisely controllable reactor addresses problems in traditional impedance-matching devices, such as large-space occupation and limited matching frequencies.

The magnetic field distribution in the orthogonal region of the high-current precisely controllable reactor used in this study is shown in Figure 5. The direction of the AC magnetic field is the same as that of magnetic core a, and the direction of the DC magnetic field is the same as those of DC magnetic cores b and c, as depicted in Figure 5. Analysis of the reactor based on Ampère’s circuital law yields Equations (3)–(9).

(3) $${N_{a}i}_{a}=H_{al}l_{al}+2H_{a2}l_{a2}+2H_{a3}l_{a2}$$

(4) $${N_{d}i}_{d}=H_{dl}l_{dl}+2H_{d2}l_{d2}\;$$

(5) $${N_{d}i}_{d}=H_{d3}l_{dl}+2H_{d4}l_{d2}\;$$

(6) $$l_{a}=l_{al}+2l_{a2}$$

(7) $$l_{d}=l_{d1}+2l_{d2}$$

(8) $$H_{a2}={\cos\alpha_{1}H}_{b}=\frac{B_{a1}}{B_{b}}H_{b}$$

(9) $$H_{a3}={\cos\alpha_{2}H}_{c}=\frac{B_{a1}}{B_{c}}H_{c}$$

In Equations (3)–(9), *N*_a_ represents the total turns of the AC winding coils composed of W_a1_ and W_a2_, or W_a3_ and W_a4_, while *N*_d_ represents the total turns of the DC winding coils on core b or core c, and *i*_a_ and *i*_d_ denote the currents that pass through the AC and DC winding coils, respectively. *H*_a1_ represents the magnetic field strength of the non-orthogonal part of the AC magnetic core, and *H*_a2_ and *H*_a3_ represent the magnetic field strengths of the two orthogonal parts of the AC magnetic core. *H*_d1_ and *H*_d2_ represent the magnetic field strengths of the non-orthogonal and orthogonal parts of DC magnetic core b, and *H*_d3_ and *H*_d4_ represent the magnetic field strengths of the non-orthogonal and orthogonal parts of DC magnetic core c. *H*_b_ and *H*_c_ represent the vector sum of the AC and DC components of the orthogonal parts of the magnetic field strengths for the AC magnetic core and DC magnetic cores b and c, respectively. Further, *l*_a_, *l*_a1_, and *l*_a2_ denote the total magnetic path length, non-orthogonal part magnetic path length, and individual orthogonal part magnetic path length of the AC magnetic core, respectively. Owing to the identical structures of the DC magnetic cores, *l*_d_, *l*_d1_, and *l*_d2_ indicate the total magnetic path length, non-orthogonal magnetic path length, and individual orthogonal magnetic path length of the two DC magnetic cores, respectively. *B*_a1_, *B*_d1_, and *B*_d2_ represent the AC and DC magnetic induction intensity components, respectively. *B*_b_ and *B*_c_ represent the vector sum of the AC and DC components of the orthogonal parts of the magnetic induction intensity for the AC magnetic core and DC magnetic cores b and c, respectively. α_1_ and α_2_, respectively, represent the angles between *B*_a1_ and *B*_b_, and between *B*_a1_ and *B*_c_. The controllable reactor designed in this study operates at a peak current of only 4 A and features an AC magnetic circuit length of 380 mm. During the production of the prototype, slight air gaps were inevitably trapped within the two AC magnetic cores, resulting in an initial magnetic permeability of *μ*_i_ = 1510. Therefore, the magnetic field intensity in the circuit is lower than the saturation magnetic field intensity *H_s_*, and the magnetic permeability exhibits good linearity. As the operating current in the controllable reactor increases, the air gap in the AC magnetic core can be widened to avoid saturation of the magnetic core, as illustrated in Figure 5, ensuring that the magnetic field strength generated by the working current remains below *H_s_* [25]. Based on the relationship B=μ·H between the magnetic field strength *H*, magnetic induction intensity *B*, and magnetic permeability *μ*, combined with Equations (8) and (9), Equation (3) can be derived as Equation (10).

(10) $$N_{a}i_{a}=H_{al}l_{al}+2\frac{B_{a1}}{B_{b}}H_{b}l_{a2}+2\frac{B_{a1}}{B_{c}}H_{c}l_{a2}\;=\frac{B_{al}}{\mu_{al}}l_{al}+2\frac{B_{a1}}{\mu_{b}}l_{a2}+2\frac{B_{a1}}{\mu_{c}}l_{a2}$$

where *μ*_a1_ is the magnetic permeability of the non-orthogonal part of the AC magnetic core. Referring to Figure 5, *μ*_b_ and *μ*_c_ represent the permeabilities of the orthogonal parts. Due to their anisotropic nature, the cores’ magnetization properties vary depending on the direction, with the material exhibiting better magnetization properties and, consequently, higher permeability in the rolling direction [26,27]. Subsequently, substituting the relationships *ϕ* = *B∙S*, where *ϕ* denotes the magnetic flux and *S* indicates the cross-sectional area, and *Li* = *N*Φ, where *L* denotes the inductance, *N* indicates the number of turns, *i* indicates the current, and Φ denotes the magnetic flux, into Equation (10) yields Equation (11).

(11) $$L=\frac{{N_{a}}^{2}}{\frac{l_{a1}}{\mu_{a1}\cdot S_{o}}+\frac{2\cdot l_{a2}}{\mu_{b}\cdot S_{o}}+\frac{2\cdot l_{a2}}{\mu_{c}\cdot S_{o}}}$$

where *S*_o_ represents the size of the orthogonal cross-sectional area. When *B_d_*_1_ and *B*_d2_ are both zero, indicating that the DC winding coils are not energized, *B*_b_ and *B*_c_ align with the material’s rolling direction, resulting in maximum *μ*_b_ and *μ*_c_. When *μ*_b_ = *μ*_c_ = *μ*_a1,_ permeabilities *μ*_b_ and *μ*_c_ reach their maximum values, in turn, to the maximum inductance *L,* as shown in Equation (12).

(12) $$L_{max}=\frac{\mu_{al}S_{o}N_{a}^{2}}{l_{a}}$$

As the direct current in the control winding I_d_ increases, *μ*_b_ and *μ*_c_ gradually decrease. When the current reaches its maximum, the two inductance sets attain their minimum. By designing the two sets of operational windings with such a number of turns that the inductance L1 induced by W_a3_ and W_a4_ is approximately equal to the minimum inductance L2 induced by W_a1_ and W_a2_, where *N*_L1_ = 24 turns for L1 and *N*_L2_ = 12 turns for L2, and alternately connecting them to the working circuit during control, the inductance range for the high-current precisely controllable reactor can be further expanded. By substituting *μ*_i_, N_L1_, and N_L2_, the maximum theoretical values of L1 and L2 obtained from Equation (12) are 1.80 mH and 0.45 mH, respectively.

**Figure 6:**
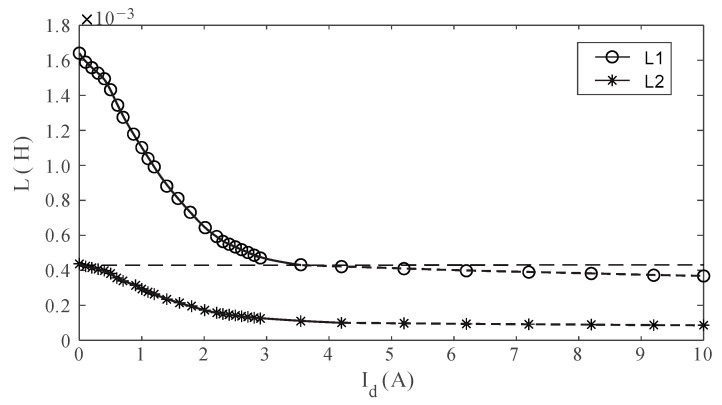
Measured variation in the inductance of the high-current precisely controllable reactor.

By setting the total turns of the DC winding coils *N*_d_ to 60 and adjusting the direct current I_d_ in the control windings, the actual variation in inductances L1 and L2 can be measured. As observed in Figure 6, the maximum values of L1 and L2 are 0.44 mH and 1.66 mH, respectively, with corresponding errors of 2.3% and 8.4% when compared to the theoretical calculation. Likewise, the minimum values of L1 and L2 are 98 μH and 0.36 mH, respectively. Thus, the overall variation of the high-current precisely controllable reactor ranges from 98 μH to 1.66 mH, equivalent to a total inductance variation of 94%, which fulfills the design requirements of this study. The rate of change in the inductance of the high-current precisely controllable reactor gradually decreases as the control current increases. Therefore, for practical use, only the portion with a relatively high rate of change is utilized, as shown by the solid line in Figure 6.

### 3.2. Analysis and Design of Resonant Capacitor

Once the range of the inductance values for the high-current precisely controllable reactor was determined, the subsequent step involved determining the capacitance values of the resonant capacitors to achieve impedance matching. As discussed above, one must ensure that at multiple frequencies within the bandwidth, the imaginary parts of *L*_p_ and *Z*_e1_ shown in Figure 4b can mutually cancel each other, that is, satisfy Equation (13), thereby ensuring that the impedance of the transmission circuit becomes resistive. Experimental measurements showed that the equivalent leakage inductance of the primary and secondary windings, referred to as the primary of the high-frequency transformer (HFT), was 3.93 µH. The equivalent inductance of the transmission cable was 217 µH, and the maximum and minimum values of the inductance of the high-current precisely controllable reactor were 1.66 mH and 0.11 mH, respectively. The turn ratio of the high-frequency transformer was 1:6 (n = 1/6).

(13) $$\left(\left(L_{t}+L_{c}\right)\cdot 2\pi f_{c}-\frac{1}{2\pi f_{c}C_{t}}\right)n^{2}-2\pi f_{c}L_{p}=0$$

where *L*_p_ represents the sum of the leakage inductances of the primary and secondary windings of the high-frequency transformer; *C*_t_ and *L*_t_ denote, respectively, the resonant capacitor and the high-current-transmission controllable reactor selected at a specific frequency *f*_c_; and *R*_c_ and *L*_c_ denote the equivalent resistance and inductance of the transmission cable, respectively. When *f*_c_ reaches its minimum value and *L*_t_ reaches its maximum value, the selected *C*_t_ for this configuration can be obtained by using Equation (13). Substituting *C*_t_ and the minimum value of *L*_t_ into Equation (13) yields the maximum frequency at which this resonant capacitor can operate. This process provides all the required resonant capacitance values for the frequency range of 10–120 kHz. Combined with the selection criteria, the resonant capacitances listed in Table 2, when paired with a controllable reactor, can achieve resistive impedance in the transmission circuit. Therefore, when the transmitting cable must be replaced, only the corresponding resonant capacitor group needs to be replaced according to the length and material of the cable, which can be adapted to different transmitting circuits, thus improving the adaptability of the device to different transmitting environments.

### 3.3. Circuit Structure Analysis and Design of the High-Frequency, High-Current Transmission Technique Based on Adaptive Impedance Matching

**Figure 7:**
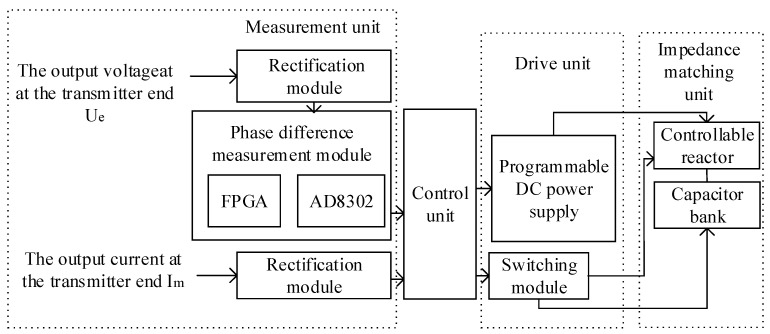
Schematic diagram of the high-frequency, high-current transmission circuit based on adaptive impedance matching.

The circuit structure of the proposed high-frequency, high-current transmission technique based on adaptive impedance matching is illustrated in Figure 7. It primarily comprises six parts: a measurement unit, a control unit, a drive unit, an impedance-matching unit, and a high-frequency transformer. The measurement unit is responsible for measuring the transmission frequency in the circuit and the phase difference between the output voltage and output current at the transmitter end. It includes a high-precision current sensor, rectification module, voltage reduction module, and phase-difference measurement module. The phase-difference measurement module includes an AD8302 and a Field-Programmable Gate Array (FPGA), which measure the phase difference between the output voltage and output current at the transmitter end. The control unit is responsible for receiving the results from the measurement module, processing them, and controlling the drive unit accordingly. The drive unit includes a switching module for selecting the capacitor and controllable reactor corresponding to the transmission frequency, as well as a programmable DC power supply to control the reactor inductance. The switching module comprises MOS transistors and electromagnetic relays that effectively separate weak electrical control from strong electrical operation. A programmable DC power supply is connected to the reactor control windings. It enables the control unit to regulate the output current to a specific magnitude, thus achieving controlled variations in the high-current precisely controllable reactor. The impedance-matching unit contains resonant capacitors and a controllable reactor, forming a resonant network with capacitive and inductive components in the transmission loop, such as a transmission cable and high-frequency transformer, to achieve frequency selection for the transmitted signal.

A high-frequency transformer (HFT), a crucial magnetic component in the circuit, provides electrical isolation of the primary and secondary sides and regulates the voltage throughout the circuit [28,29]. Compared with traditional transformers, HFTs exhibit significant advantages in terms of performance efficiency, manufacturing costs, transportation, and installation. Therefore, they are widely employed in applications in which stringent requirements for transformer volume and weight exist. Currently, they are extensively utilized in emerging areas such as new energy, power grid systems, and electric vehicles, indicating a new trend of HFTs replacing conventional low-frequency transformers. The HFT effectively amplifies the AC signal that undergoes frequency selection through the RLC resonance circuit by increasing the transmitted voltage connected to the transmission cable. This directional amplification enhances transmission power while reducing the DC voltage supplied to the transmitter by the front stage.

#### 3.3.1. Analysis and Design of the Phase Difference Measurement Module

The phase-difference measurement module determines whether the transmission circuit is in a resonant state and whether its impedance is resistive by measuring the phase difference between the output voltage and the current at the transmitter. In this study, AD8302 from Analog Devices was chosen for its capability to measure the gain (amplitude ratio) and phase difference simultaneously between two input signals over a frequency range from low frequency to 2.7 GHz. Only the phase difference measurement function of the AD8302 was utilized in this study, with a measurement range from –180° to 180°, corresponding to an output voltage range from 0 V to 1.8 V. The output voltage sensitivity is 10 mV/°, with a measurement error of less than 0.5°. The conversion rate for the phase output is 30 MHz, and the response time ranges from 40 ns to 500 ns, satisfying the measurement requirements. The AD8302 effectively converts angular values that make it challenging for the control unit to handle voltage values for easier processing.

**Figure 8:**
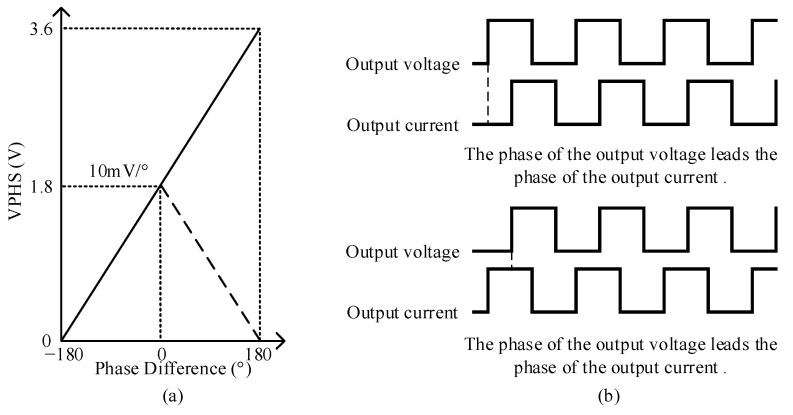
(**a**) Ideal phase response curve. (**b**) Waveforms of the output voltage and output current at different phases.

The AD8302 covers the same voltage range when measuring from –180° to 0° and from 0° to 180°, with opposite slopes as depicted by the dashed line in Figure 8a, illustrating the ideal response curve of the phase. Therefore, accurately determining the phase relationship between the output voltage and current is challenging, which affects the control of the variable reactor when only the AD8302 is used. To address this limitation, an FPGA was employed to measure the positive and negative phase difference angles. The control unit processes the voltage *V*_PHSm_ from the *V*_PHS_ pin of the AD8302 by reading the measurement results from the FPGA. This process ensures that the phase response curve of the phase difference measurement module becomes linear. The relationship between the phase difference angle and *V*_PHS_ is depicted by the solid line in Figure 8a. This implementation achieves a one-to-one correspondence between the phase-difference angle and voltage *V*_PHS_, effectively resolving the problem of imprecise measurement of the phase relationship between the output voltage and output current at the transmitter.

To analyze the phase relationship between the output voltage and output current, different phase waveforms of the output voltage and output current are illustrated in Figure 8b. When the rising edge of the output voltage occurs and the emitter current is low, the output voltage leads to an output current, indicating an inductive circuit. Conversely, if the output current is high, the output voltage lags the output current, suggesting a capacitive circuit.

**Figure 9:**
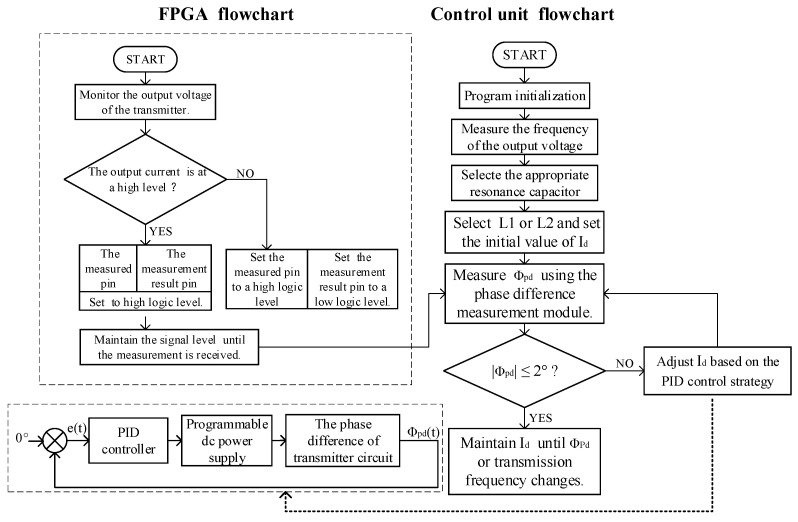
Control unit and FPGA control flowcharts.

Therefore, the FPGA control flowchart is depicted in Figure 9. Upon receiving the signal to start the measurement from the control unit, the level of the output current is examined when a rising edge in the output voltage is detected. If the output current is high, the measured pin is set to a high level, and the measurement result pin is set to a high level. If the output current is low, the measured pin is set to a high level, and the measurement result pin is set to a low level. When the control unit sends a signal to end the measurement phase difference, both the measured pin and the measurement result pin are set to low levels. Otherwise, the level signals of these pins are maintained until the signal to end the measurement is received.

**Figure 10:**
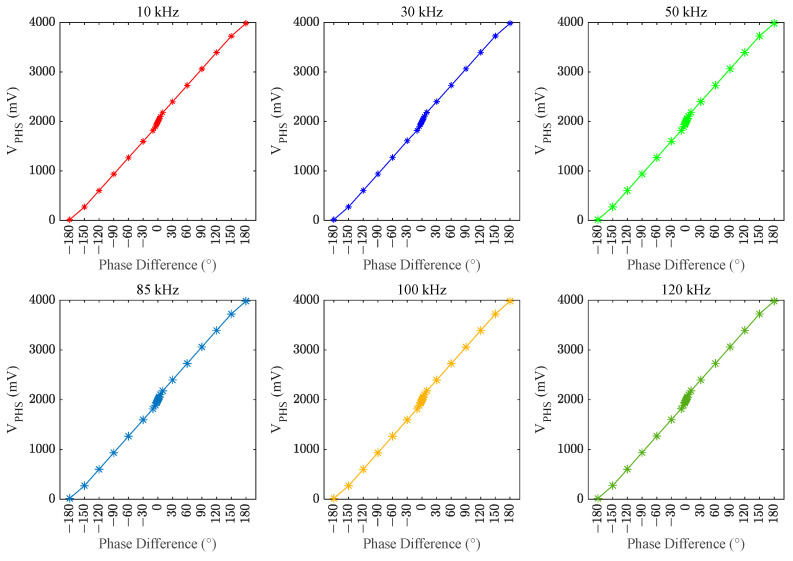
Measured phase response curves of the phase-difference measurement module.

The control module processes the voltage *V*_PHSm_ and the level signal from the measurement result pin, output by the phase-difference detection module composed of the AD8302 and FPGA, to obtain the voltage *V*_PHS_. This process enables the detection of the actual phase difference between the output voltage and output current at that moment. The measured phase response curve of the phase-difference measurement module is illustrated in Figure 10. Although slightly different from the ideal phase response curve, the phase difference angle and *V*_PHS_ therein are essentially linear. In addition, the measured phase response is highly consistent at different frequencies, which satisfies the requirements of this study.

#### 3.3.2. Basic Control Flow of the Control Unit

To process the data from the measurement unit and make corresponding controls on the driving unit, a control flowchart of the control unit based on a high-frequency, high-current transmission technique is illustrated in Figure 9. Initially, the resonant capacitor and controllable reactance inductor (*L*1 or *L*2) must be selected based on the measured transmission frequency, as calculated in the previous section. Subsequently, the phase difference between the output voltage and output current at the transmitter end is detected. A signal to start measuring the phase difference is sent to the FPGA. Upon detecting that the measured pin is at a logically high level, the level of the measurement result pin from the FPGA is read. Additionally, the *V*_PHSm_ of the *V*_PHS_ pin of the AD8302 is collected through the ADC. Once the collection is complete, a signal is sent to the FPGA to end the measurement. The control unit initially applies Kalman filtering to filter *V*_PHSm_, reducing errors. The phase difference was determined by combining the FPGA measurement results. When the FPGA measurement result pin is at a high level, the voltage signal is directly used to determine the phase difference. When the FPGA measurement result pin is at a low level, the voltage processing is performed using *V*_PHS_ = (*V*_ZPD_ + *V*_PHSm_), ensuring a one-to-one correspondence between the phase difference angle and voltage information. The processed voltage *V*_PHS_ is then used to determine the phase-difference angle at that moment, where *V*_ZPD_ represents the voltage information when the phase-difference angle is 0°. When the absolute value of the phase-difference angle is greater than 2°, a proportional-integral-derivative (PID) controller is employed to adjust the inductance value of the controllable reactance, ensuring that the circuit exhibits resistive characteristics. The error (*e*(*t*)) between the measured phase difference Φ_pd_(*t*) and 0° at this point is used as an input into the PID controller. According to the PID control strategy, the control winding current of the high-current precisely controllable reactor is adjusted to change its inductance, thereby controlling the phase difference between the circuit voltage and current to maintain a resistive circuit. When the phase-difference angle is less than 2°, the control winding current is maintained. The phase difference must be measured when the circuit reaches a steady state during adaptive impedance matching using the controllable reactor. The resulting average adjustment time is approximately 4 s. During operation, the transmission frequency and phase difference between the output voltage and current at the transmitter end must be monitored continuously. If the transmission frequency changes, whether a need exists to change the resonant capacitor must be checked promptly. Timely adjustments to the control winding current are required when the phase-difference angle exceeds 2°.

## 4. Experimentation and Analysis

**Figure 11:**
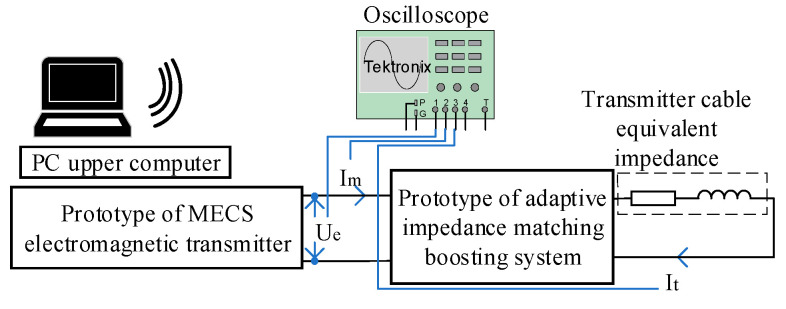
Schematic of the prototype test.

**Figure 12:**
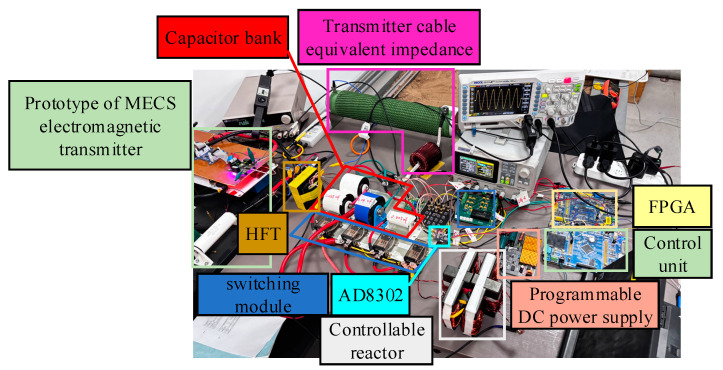
Experimental circuit and on-site wiring of the prototype.

To further analyze the feasibility of the designed structure and control methods employing the proposed high-frequency, high-current transmission technique based on adaptive impedance matching, the design and assembly of a low-power prototype for indoor testing are herein described. In accordance with project requirements, a 217 µH fixed inductor and a 25 Ω high-power resistor were utilized to simulate collectively the inductance and resistance generated when laying a 100 m transmission cable during outdoor exploration. This simulation also considers the grounding resistance generated by the transmission electrode and the earth. Figure 11 shows a schematic of the test prototype, whose experimental circuit and on-site wiring are illustrated in Figure 12. To ensure the safety of the indoor testing, the output voltage of the transmitter was maintained at approximately 25 V, and the transmitter was controlled to transmit at different frequencies as required by the project. Additionally, the output voltage (*U*_e_), current (*I*_m_) at the transmitter end, and transmission current (*I*_t_) in the transmission circuit were continuously measured.

**Figure 13:**
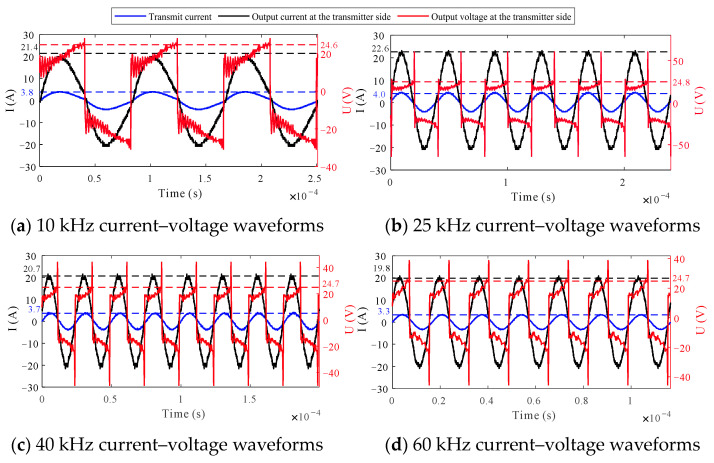

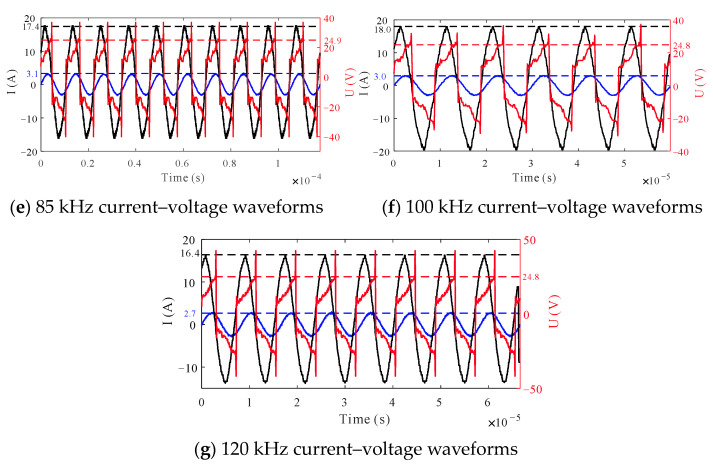
Measured current–voltage waveforms.

Analyzing Figure 13 reveals that the transmission current waveform becomes a sinusoidal wave after impedance matching, with maximum values consistently exceeding 2.7 A. The output voltage at the transmitter is in phase with the output current.

**Figure 14:**
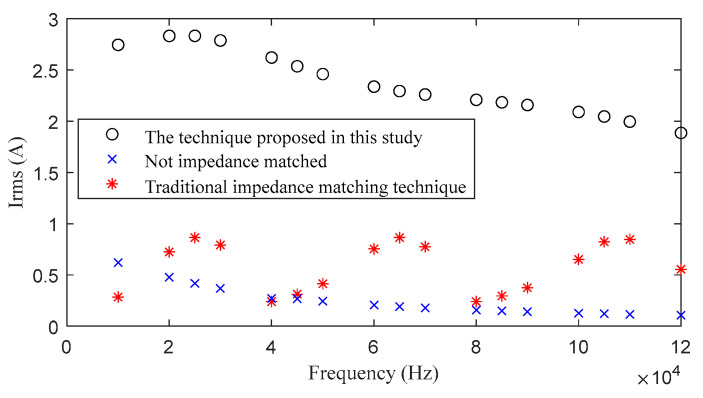
Comparison of the transmission current obtained using the high-frequency, high-current transmission technique and those resulting from other methods.

Figure 14 compares the currents in the transmission circuit and the computational transmission currents obtained when impedance matching is or is not implemented. The output voltage at the transmitter end and the impedance of the transmission circuit remain constant during both experiments and computations. When excluding impedance matching, the transmitter is directly connected to the transmission cable. When computing the transmission current using the traditional impedance matching technique, as shown in Figure 2, which uses only resonant capacitors to match the impedance, the number of selected resonant capacitors should remain consistent with the prototype. Utilize Multisim 14.0 to compute the fundamental-frequency transmission current after integrating capacitors of 0.18 μF, 27.5 nF, and 10 nF into the transmission circuit, respectively. The results in Figure 14 reveal that without impedance matching, the transmission current rapidly decreases, rendering it unsuitable to meet the detection requirements. In traditional impedance matching, the effectiveness of the effective value of the fundamental-frequency transmission current is significant only near the resonance frequency of the resonant capacitor and circuit inductance. However, because of the limited number of resonant capacitors, the enhancement of the transmission current for multiple frequencies under wideband conditions becomes less satisfactory as the frequency bandwidth increases. Incorporating additional resonant capacitors would increase the volume and mass of the instrument.

However, with the proposed high-frequency, high-current transmission technique based on adaptive impedance matching, the transmission current is significantly enhanced compared to scenarios without impedance matching and those using only traditional impedance matching. Under the same transmission voltage, compared to the scenario without impedance matching in the transmission circuit, the maximum increase in the transmission current reaches 16.7 times, with an average improvement of 10.8 times. Compared to using only traditional impedance-matching techniques in the transmission circuit, the maximum increase in the transmission current reaches 10.0 times, with an average improvement of 4.2 times. These findings clearly demonstrate the effectiveness and superiority of the high-frequency, high-current transmission technique.

## 5. Conclusions

To address the challenge of large current excitation at multiple frequencies under wide-frequency conditions, we introduced a high-frequency, high-current transmission technique for high-resolution multiple earth electrical characteristic measurement systems (MECS) based on adaptive impedance matching. By integrating resonant capacitors, controllable reactors, and the corresponding control circuits, the proposed technique achieves frequency selection for the transmission signal. A high-current precisely controllable reactor with a wide range of variation (from 98 μH to 1.66 mH) was designed, achieving a total inductance variation range of 94%, which reduces both the number of resonant capacitors and the size of the instrument. Moreover, the adaptability of the device to different transmitting circuits can be significantly improved by replacing a few resonant capacitor packs. The use of a high-frequency transformer enables directional amplification of the transmission current, overcoming the challenge of increasing the voltage in the pre-stage DC-DC converter. The achieved enhancement in the transmission current for multiple frequencies within the range of 10–120 kHz resulted in a peak transmitted active power of 200 W. Under the same transmission voltage, compared to the transmission circuit without impedance matching, the transmission current increased by a maximum of 16.7 times, with an average improvement of 10.8 times, whereas compared to the transmission circuit using only traditional impedance matching, the transmission current increased by a maximum of 10.0 times, with an average improvement of 4.2 times. Thus, the proposed high-frequency, high-current transmission technique effectively improved the efficiency and resolution of shallow subsurface target exploration. However, the technique’s underlying principles limit the attainable current increase in the time-domain electromagnetic method (TEM). Stably increasing the energy of all frequency signals is not possible when transmitting multiple-frequency signals, and it may even suppress signals at certain frequencies. Nevertheless, adjusting the control strategy of the controllable reactor enables the tuning of the transmission circuit’s reactance, thereby maximally reducing the impedance of the circuit when transmitting multiple-frequency signals, which, in turn, increases the transmission current of the multi-frequency signals.

## Figures and Tables

**Table 1:** Comparative analysis between techniques for enhancing the intensity of artificial high-frequency field sources.

Solution	Boosting Method	Circuit Impedance Reduction Method	Frquency	Strengths	Limitations
Xiaohua et al. [8]	Transmission power increases	——	0.1 Hz–10 kHz	48 kW transmission power, 950 V max. voltage	Heavy, complex, costly
Qingyun et al. [9]	Wire structure optimization	Wire structure optimization	——	Increased by 1 A at 8533 Hz	Customization required
Qihui et al. [10]	Transmission cable impedance reduction	Multiple electrodes and multi-strand or thicker cables	0.1 Hz–10 kHz	All frequencies are boostable	Increased field workload
Streich et al. [11]	Establishment of multi-polarization fields	——	1/1024 s–8.2 kHz	Good subsurface coverage, enhanced illumination	Higher demands on instrument precision, quantity, and control
Meng et al. [12]	Multi-directional source synchronous transmission	——	0.1 Hz–10 kHz	Boosted electromagnetic strength	
Qihui et al. [13]	Double transmitter system	——	0.1 Hz–10 kHz	Increased by 1 time at 10 kHz	
Qingbin et al. [14]	RLC series resonance	Resonant capacitors	10 kHz–200 kHz	Boosted specific frequency transmission	Limited to multiple frequencies
Dang et al. [15]			59 Hz–8.8 kHz	Maximum increase of 10.5 times, average of 4.1 times	
Technique proposed in this study	Adaptive impedance matching through phase comparison	Resonant capacitors, high-current precisely controllable reactor, HFT, etc.	10 kHz–120 kHz	Higher and more frequency, Maximum increase of 16.7 times, average of 10.8 times, easy use	Limited in TEM and multi-frequency signal

**Table 2:** Selection of resonant capacitors and controllable reactors in the range 10–120 kHz.

Frequency (Hz)	Impedance Matching Unit	
	Resonant Capacitor	Controllable Reactor
10,000 ≤ fc < 16,700	0.14 µF	*L*2
16,700 ≤ fc < 23,500		*L*1
23,500 ≤ fc < 39,500	0.025 µF	*L*2
39,500 ≤ fc < 55,500		*L*1
55,500 ≤ fc < 88,000	0.005 µF	*L*2
88,000 ≤ fc < 120,000		*L*1

## Data Availability

Data are contained within the article.
